# Supplementary material for: Structural insights into FSP1 catalysis and ferroptosis inhibition
Source: Nat Commun. 2023 Sep 22;14:5933. doi: 10.1038/s41467-023-41626-7 (PMC10516921; doi:10.1038/s41467-023-41626-7)
Supplement: Supplementary file 1 — Supplementary information [file 41467_2023_41626_MOESM1_ESM.pdf]

SUPPLEMENTARY INFORMATION

Structural insights into FSP1 catalysis and ferroptosis inhibition

Yun Lv[1#], Chunhui Liang[2#], Qichao Sun[1#], Jing Zhu[3#], Haiyan Xu[1], Xiaoqing Li[1], Yao-yao Li[4], Qihai Wang[5*], Huiqing Yuan[1, 6*], Bo Chu[2*], Deyu Zhu[1*]

1 Department of Biochemistry and Molecular Biology, School of Basic Medical Sciences, Cheeloo College of Medicine, Shandong University, Jinan 250012, China.

2 Department of Cell Biology, School of Basic Medical Sciences, Cheeloo College of Medicine, Shandong University, Jinan 250012, China.

3 State Key Laboratory of Microbial Technology, Shandong University, Qingdao 266237, China.

4 Key Laboratory of Chemical Biology (Ministry of Education), School of Pharmaceutical Sciences, Cheeloo College of Medicine, Shandong University, Jinan 250012, China.

5 School of bioengineering, Jingchu University of Technology, Jingmen 448000,China.

6. Key Laboratory of Experimental Teratology of Ministry of Education, Institute of Medical Sciences, the Second Hospital, Cheeloo College of Medicine, Shandong University, Jinan 250031, China.

[#] These authors contributed equally to this work.

[*] To whom correspondence should be addressed: Q.W.: Ph: +86-724-2313500, Email: wangqihai@jcut.edu.cn; H.Y.: Ph: +86-531-85875118, Email: lyuanhq@sdu.edu.cn; B.C.: Ph: +86-531-88395114, Email: chubo123@sdu.edu.cn; D.Z.: Ph: +86-531-88382092-416, Email: zhudeyu@sdu.edu.cn

Table of Contents:

Supplementary Figures: 1-15

Supplementary Table 1-4

Supplementary References

Supplementary Figures

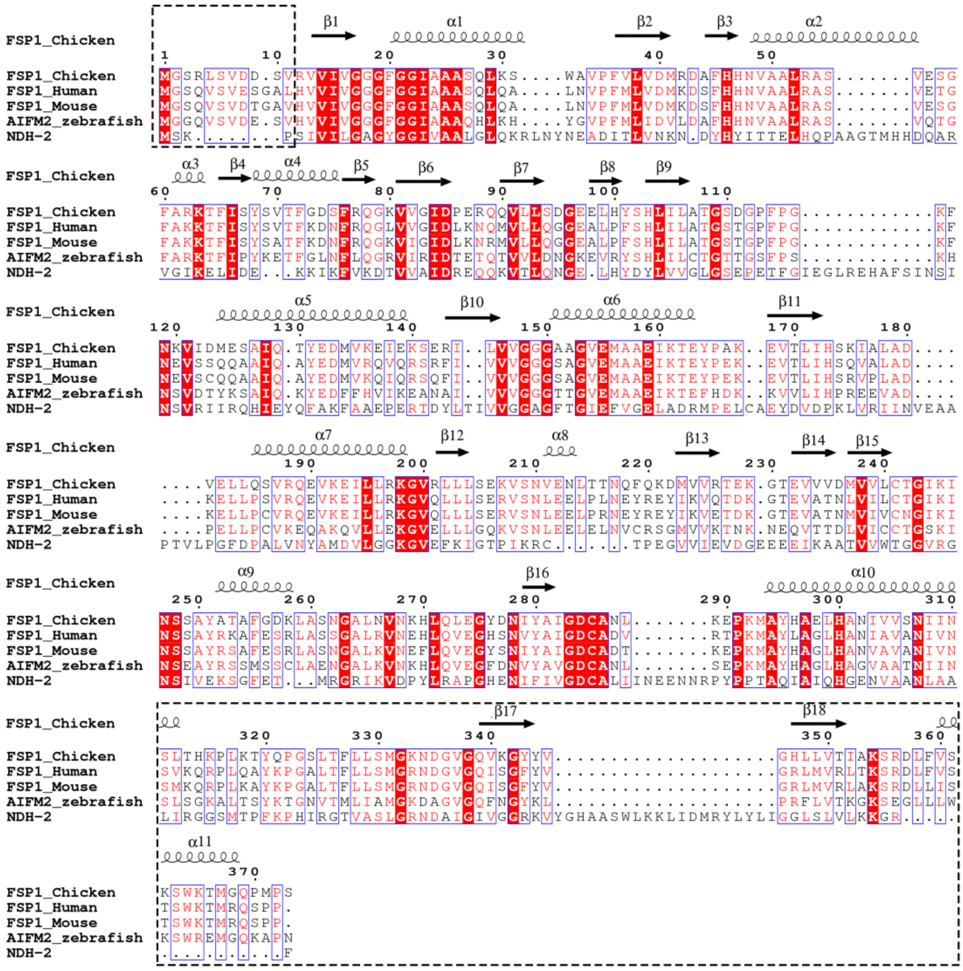

Supplementary Figure 1 Sequence alignment of cFSP1 (FSP1_chicken) (XP_040530400.1 [https://www.ncbi.nlm.nih.gov/protein/XP_040530400.1]) with FSP1_human (NP_001185625.1 [https://www.ncbi.nlm.nih.gov/protein/NP_001185625.1]), FSP1_mouse (NP_001034283.1 [https://www.ncbi.nlm.nih.gov/protein/NP_001034283.1]), AIFM2_zebrafish (NP_001186939.1 [https://www.ncbi.nlm.nih.gov/protein/NP_001186939.1]), and NDH-2 (type II NADH dehydrogenase from *Caldalkalibacillus thermarum*, WP_007502350.1 [https://www.ncbi.nlm.nih.gov/protein/WP_007502350.1]). cFSP1 shares about 70%, 69%, 68% and 25% sequence identities with FSP1_human, FSP1_mouse, AIFM2_zebrafish, and NDH-2, respectively. Numbers above the sequences are labeled based on cFSP1. Secondary structures of cFSP1 are schematically represented above the sequences. N-terminal myristoylation motif excluded in the crystallized cFSP1 and C-terminal domain (CTD) are indicated by black dashed boxes. Strictly conserved residues are marked with red background. Similar residues are shown in red color.

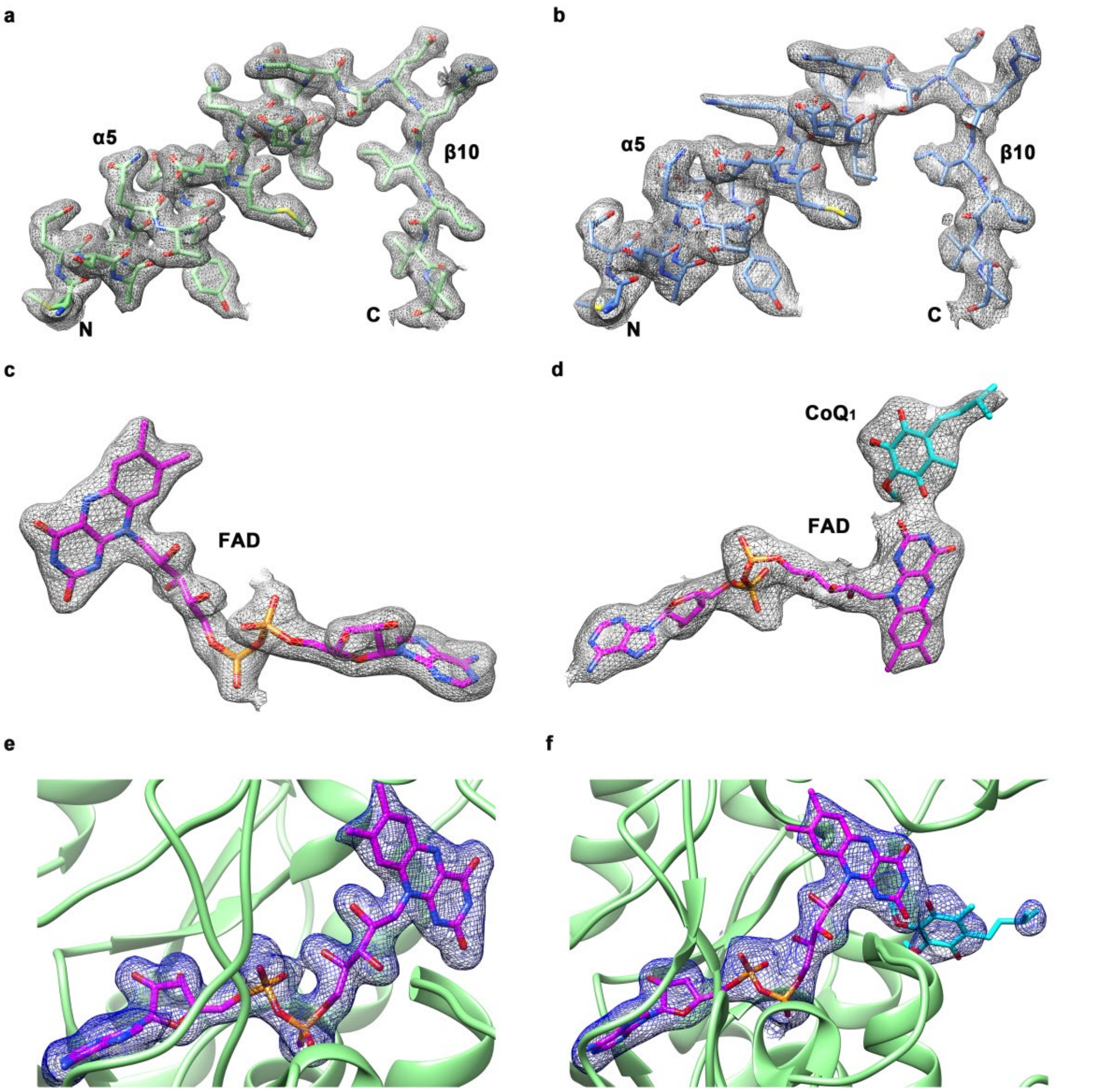

Supplementary Figure 2 Electron density maps. **a,** 2*F*o-*F*c electron density map contoured at 1.0 σ level around the residues corresponding to α5 and β10 (residues Met124 – Val147) of cFSP1$^{\Delta N}$. **b**, 2*F*o-*F*c electron density map contoured at 1.0 σ level around the residues corresponding to α5 and β10 (residues Met124 – Val147) of cFSP1$^{\Delta N}$-CoQ$_1$ complex. **c**, 2*F*o-*F*c electron density map contoured at 1.0 σ level around bound FAD of cFSP1$^{\Delta N}$. **d**, 2*F*o-*F*c electron density map contoured at 1.0 σ level around bound FAD and CoQ$_1$ of cFSP1$^{\Delta N}$-CoQ$_1$ complex. **e**, *F*o-*F*c omit map contoured at 2.0 σ level around bound FAD of cFSP1$^{\Delta N}$. **f**, *F*o-*F*c omit map contoured at 2.0 σ level around bound FAD and CoQ$_1$ of cFSP1$^{\Delta N}$-CoQ$_1$ complex.

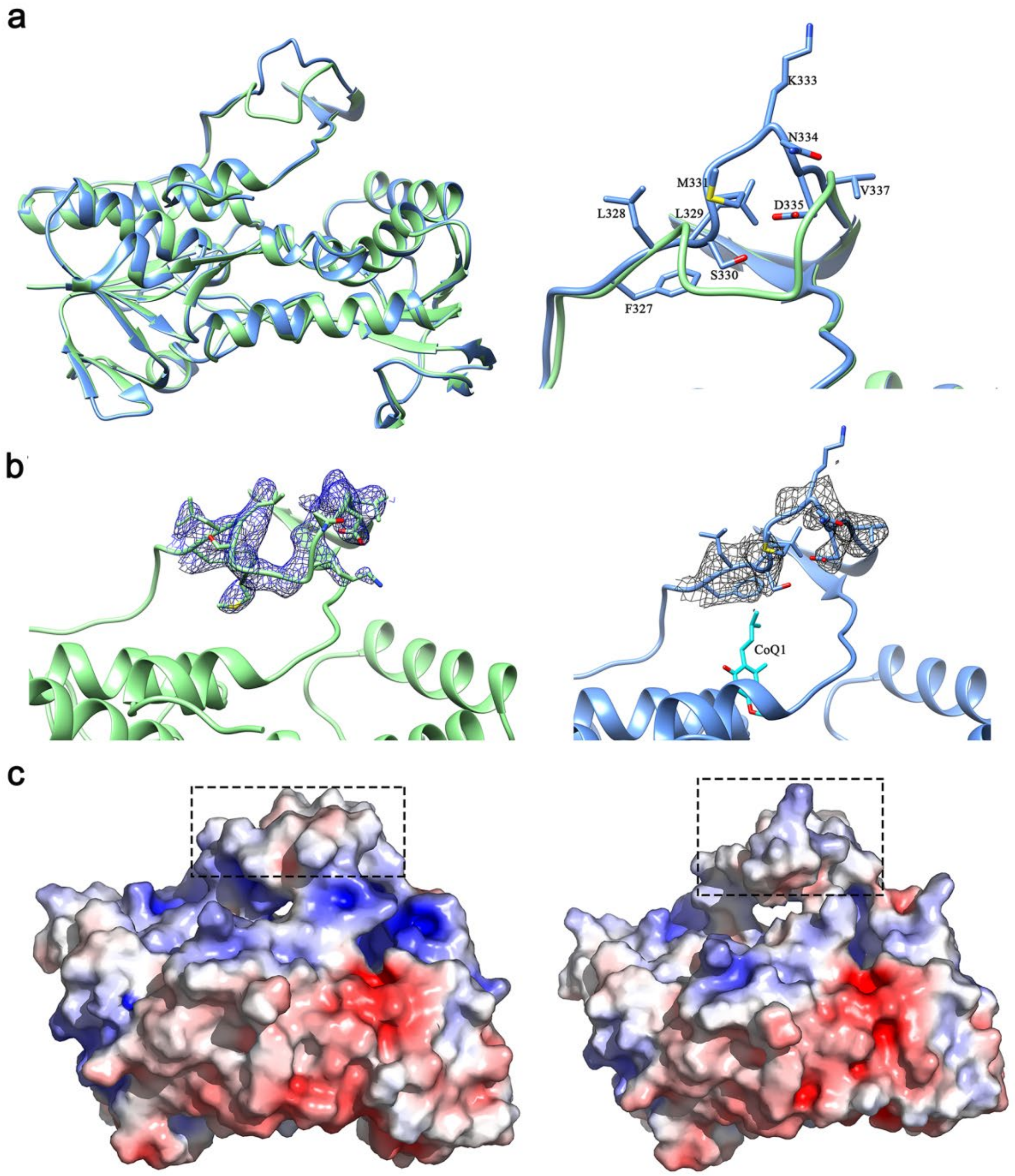

Supplementary Figure 3 Conformational changes of loop 327-337 in CTD of cFSP1$^{\Delta N}$ upon CoQ1 binging. **a,** Superposition of cFSP1$^{\Delta N}$ monomers in substrate-free (lime) and CoQ1-bound (blue) states. Right, close-up view of the conformational rearrangement of the loop 327-337. Sequence numbers are labeled based on CoQ1-bound cFSP1$^{\Delta N}$. Amino acid side chains are shown as stick models. **b,** The 2Fo – Fc electron-density maps for the loop 327-337 of substrate-free cFSP1$^{\Delta N}$ and CoQ1-bound cFSP1$^{\Delta N}$ are shown in blue and in gray (contoured at 0.6σ), respectively. **c,** CoQ1 binding also generates an obvious change in electrostatic potential surface around the loop 327-337.The electrostatic potential surfaces of cFSP1$^{\Delta N}$ monomers in substrate-free and CoQ1-bound states are shown at left and right, respectively. White, blue and red indicate neutral, positive and negative surfaces, respectively. The areas of prominent change around the loop 327-337 are indicated by black dashed box.

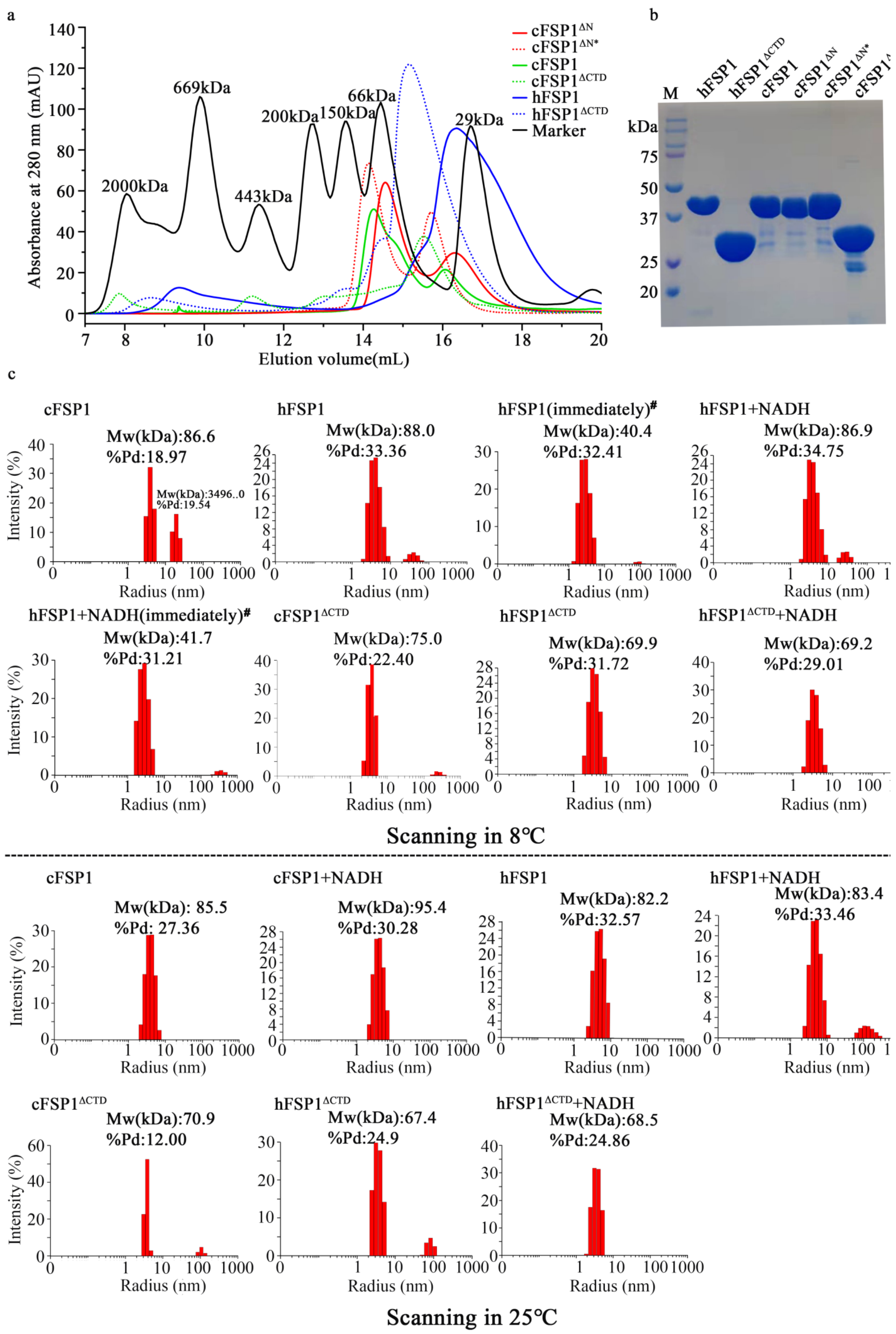

Supplementary Figure 4 Gel filtration chromatography on a Superdex 200 Increase 10/300 GL column (**a**), SDS–PAGE (**b**) and dynamic light scattering (DLS) (**c**) of FSP1 proteins. **a,** The gel filtration profiles of gel filtration molecular weight markers (Marker) and FSP1 proteins were indicated in the figure, the markers are as the follows: Carbonic Anhydrase (29 KDa), Albumin (66 KDa), Alcohol Dehydrogenase (150 KDa), β-Amylase (200 KDa), Apoferritin (443 KDa), Thyroglobulin (669 KDa),

Blue dextran (2000 KDa). The peaks of the markers were labeled with the corresponding molecular weight, cFSP1$^{\Delta N*}$ represent the methylated cFSP1$^{\Delta N}$. **b,** Lane M: marker proteins, the apparent molecular weights are indicated on the left of the panel **b**; Other lanes show the purified hFSP1 and cFSP1 proteins as indicated above, cFSP1$^{\Delta N*}$ represent the methylated cFSP1$^{\Delta N}$. **c,** DLS analysis of cFSP1 and hFSP1 with or without 25 μM NADH at 8 ℃ or 25 ℃, as indicated in the panel **c**, indication with a # represent that the purified hFSP1 is recommended to be frozen well and scanned as soon as possible after thawing, which has a relatively low reproducibility. Hydrodynamic radius of chitosan measured by DLS (DynaPro NanoStar, Wyatt Technology, Santa Barbara, CA, USA).The experiments in **a** and **b** were perfomed only once. Data in **c** were independenty repeated twice with similar results. Source data are provided as a Source Data file.

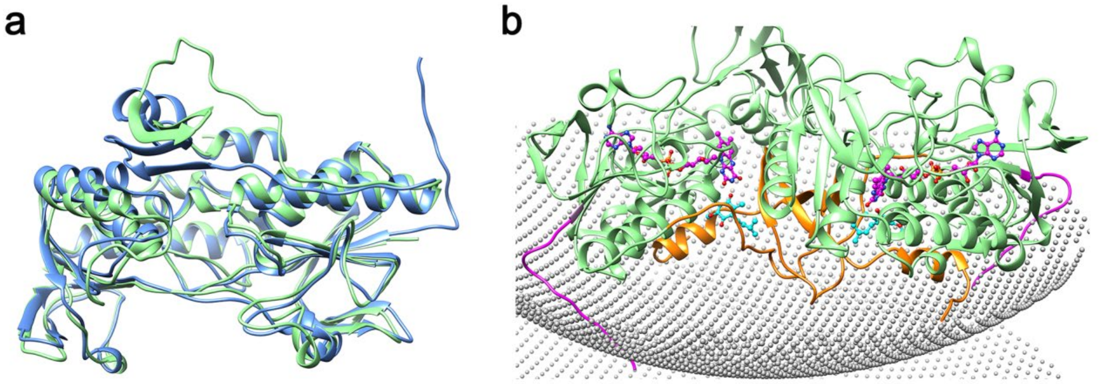

Supplementary Figure 5 The CTD may also be involved in membrane association. **a,** Structural comparison of cFSP1$^{\Delta N}$ (lime, CoQ1-bound form) with AlphaFold model[1] (blue) reveals that the major structural difference occurs in the CTD. **b,** The cartoon representation shows the spatial position of cFSP1 dimer in a double-membrane (grey spheres) that was calculated by using PPM server (https://opm.phar.umich.edu/ppm_server3)[2]. The myristoylation motif of cFSP1 is added by hand according to the AlphaFold model and coloured in pink, CTD and remaining part of cFSP1 are coloured in orange and lime, respectively. FAD and CoQ1 is shown as ball and stick representation. In this model, two myristoylation motifs serve as membrane anchors, the residues 328-329 and 334-337 from each CTD in the dimer associate with the membrane.

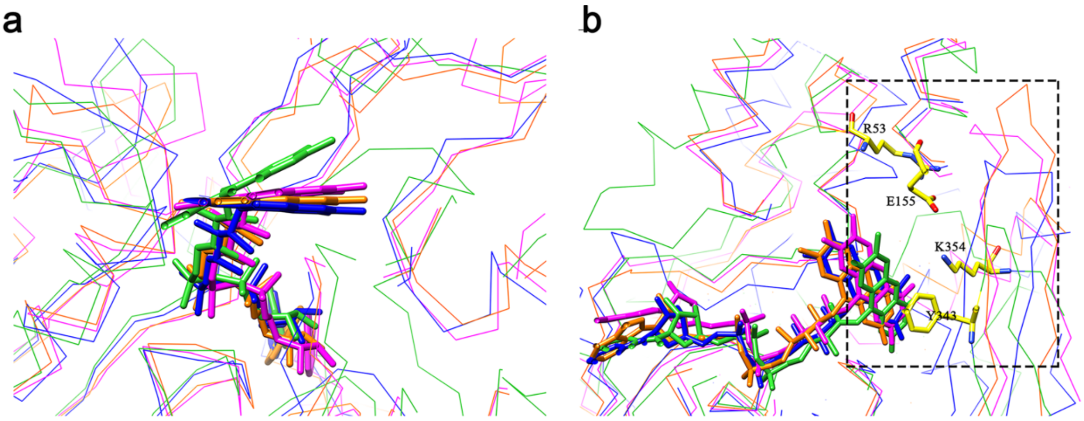

Supplementary Figure 6 The active center seems to be also a unique feature of FSP1. The structural alignment among cFSP1 (substrate-free form, green), NDH-2 (PDB 4NWZ [https://doi.org/10.2210/pdb4NWZ/pdb], pink), Ndi1 (PDB 4G6G [https://doi.org/10.2210/pdb4G6G/pdb], orange) and AIF (PDB 1M6I [https://doi.org/10.2210/pdb1M6I/pdb], blue) highlighting the unique location of the FAD isoalloxazine ring (**a**) and the FAD hydroxylation pocket (**b**) of cFSP1. **a,** FAD is shown as stick representation, FAD atoms of cFSP1, NDH-2, Ndi1 and AIF are coloured as green, pink, orange and blue, respectively. **b,** For clarity, only residues Arg53, Glu155, Lys354 and Tyr343 from the other chain of the dimer, forming the FAD hydroxylation pocket of cFSP1 are shown as green sticks. The obvious difference between cFSP1 and other proteins (NDH-2, Ndi1 and AIF) in this region is highlighted in the black dashed box.

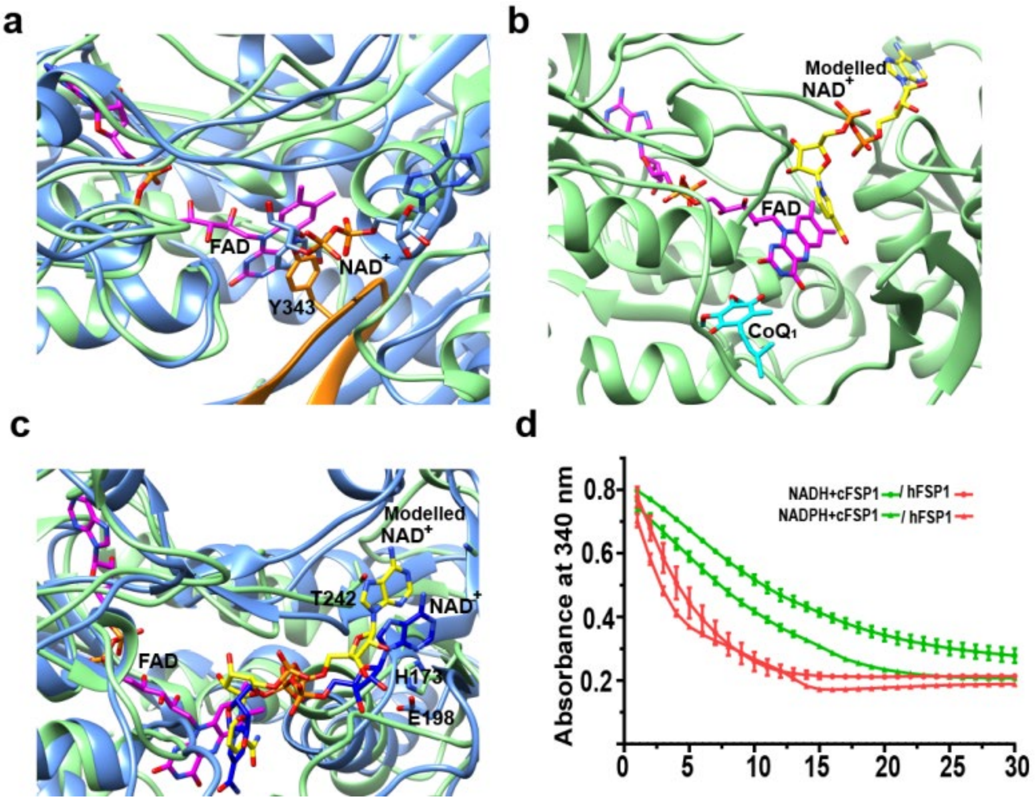

Supplementary Figure 7 Analysis of the predicted NAD(P)H-binding pocket in cFSP1. **a,** The cFSP1 (lime) NAD(P)H-binding pocket is structurally similar to the *Caldalkalibacillus thermarum* NDH-2 (blue, PDB 5KMS [https://doi.org/10.2210/pdb5KMS/pdb]) NADH-binding pocket, the position of NADH that would cause a steric clash with Tyr343 from the other chain in the dimer is highlighted in the dash box. **b,**Model of hypothetical cFSP1-NADH-CoQ1 complex, the NAD+ molecule was adapted into cFSP1 as observed in NDH-2, and the conformation of Tyr343 was adjusted by hand in order to accommodate the NAD+. **c,** The structural alignment among the modeled cFSP1-NAD+ complex (lime), NDH-2 (PDB 5KMS [https://doi.org/10.2210/pdb5KMS/pdb], blue), highlighting the different interactions between AMP portion of NADH and these proteins. The polar residues of cFSP1 close to the AMP portion, such as Thr242, Lys207 and His173, are shown as stick representation. Residue Glu198 of NDH-2 (His173 in cFSP1) forms two hydrogen bonds with two hydroxyl groups of the ribose of the AMP portion and thus determines NDH-2 selectivity for NADH over NADPH. **d,** NADH or NADPH consumption assay (340 nm) using 0.2 μM cFSP1 or hFSP1 in combination with CoQ1, data represent the mean ± s.e.m. of three experiments ($n = 3$). Source data are provided as a Source Data file.

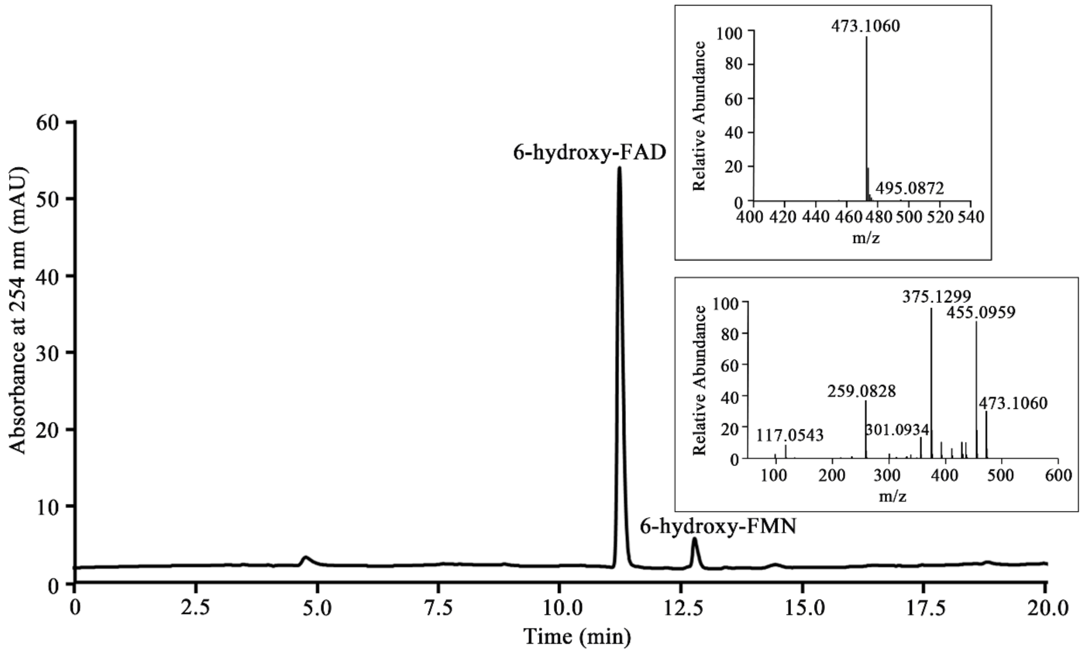

Supplementary Figure 8 HPLC-MS/MS analysis identifies 6-hydroxy-FMN as the main impurity of the purified 6-hydroxy-FAD. The upper insert panel shows the MS spectrum, the ion at m/z 473.1060 represents the $[M+H]^+$ ion of 6-hydroxy-FMN. The lower insert panel shows the tandem mass spectrum resulting from higher-energy collision dissociation (HCD) of the precursor ion ($[M+H]^+ = 473.11$). 6-hydroxy-FAD (calculated mass $[M+H]^+$, 802.1593) and 6-hydroxy-FMN (calculated mass $[M+H]^+$, 473.1068) were eluted at 11.3 min and 12.8 min, respectively. Data are available from figshare (https://doi.org/10.6084/m9.figshare.23255453).

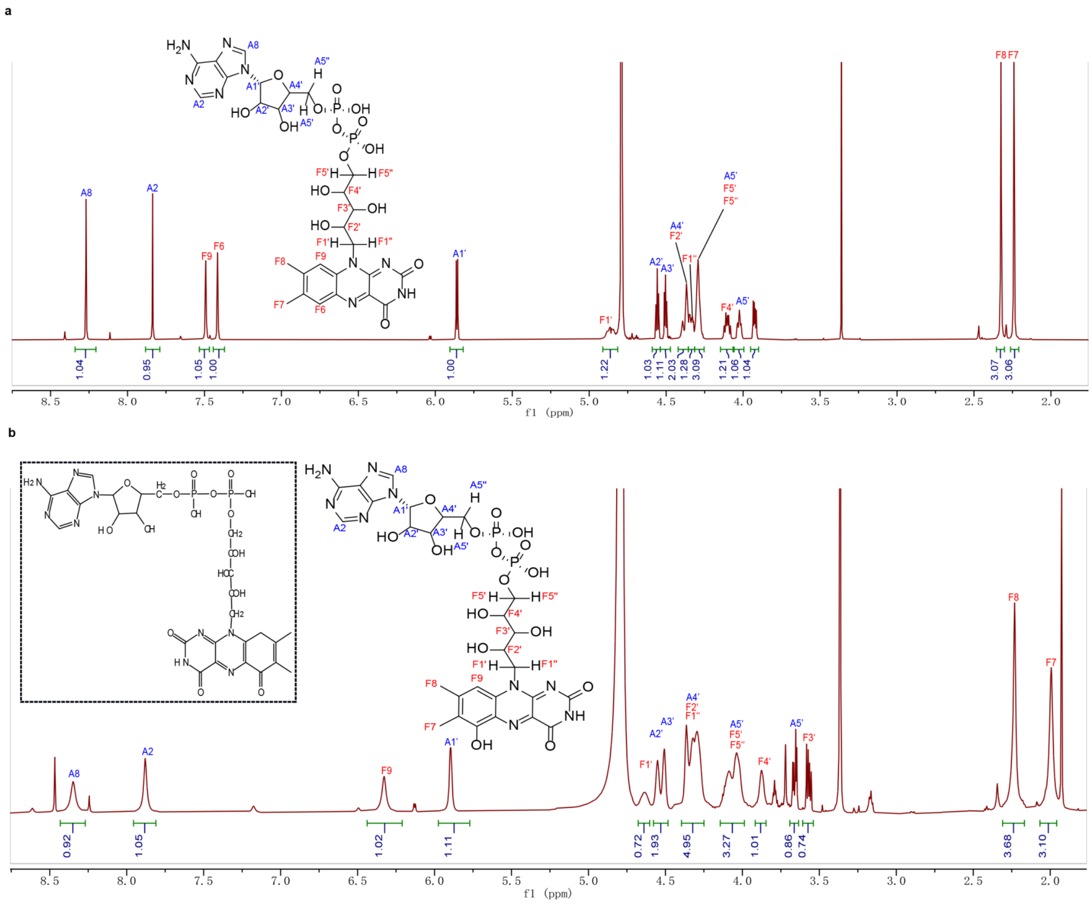

Supplementary Figure 9 The ^{1}H NMR spectra of FAD standard (**a**) and the purified 6-hydroxy-FAD (**b**) were measured on a Bruker Avance DRX-600 spectrometer in D_2O (with TMS as the internal standard). The proton peaks in the spectra were assigned and labeled according to the chemical structure in the insert panels. In the lower part, two insert panels show the chemical structure of two isomeric forms of 6-hydoxy-FAD (in the free acid form). The lower insert should be the right one, and another isomer including ketonyl at the 6 position is highlighted in the dashed box and may be ruled out.

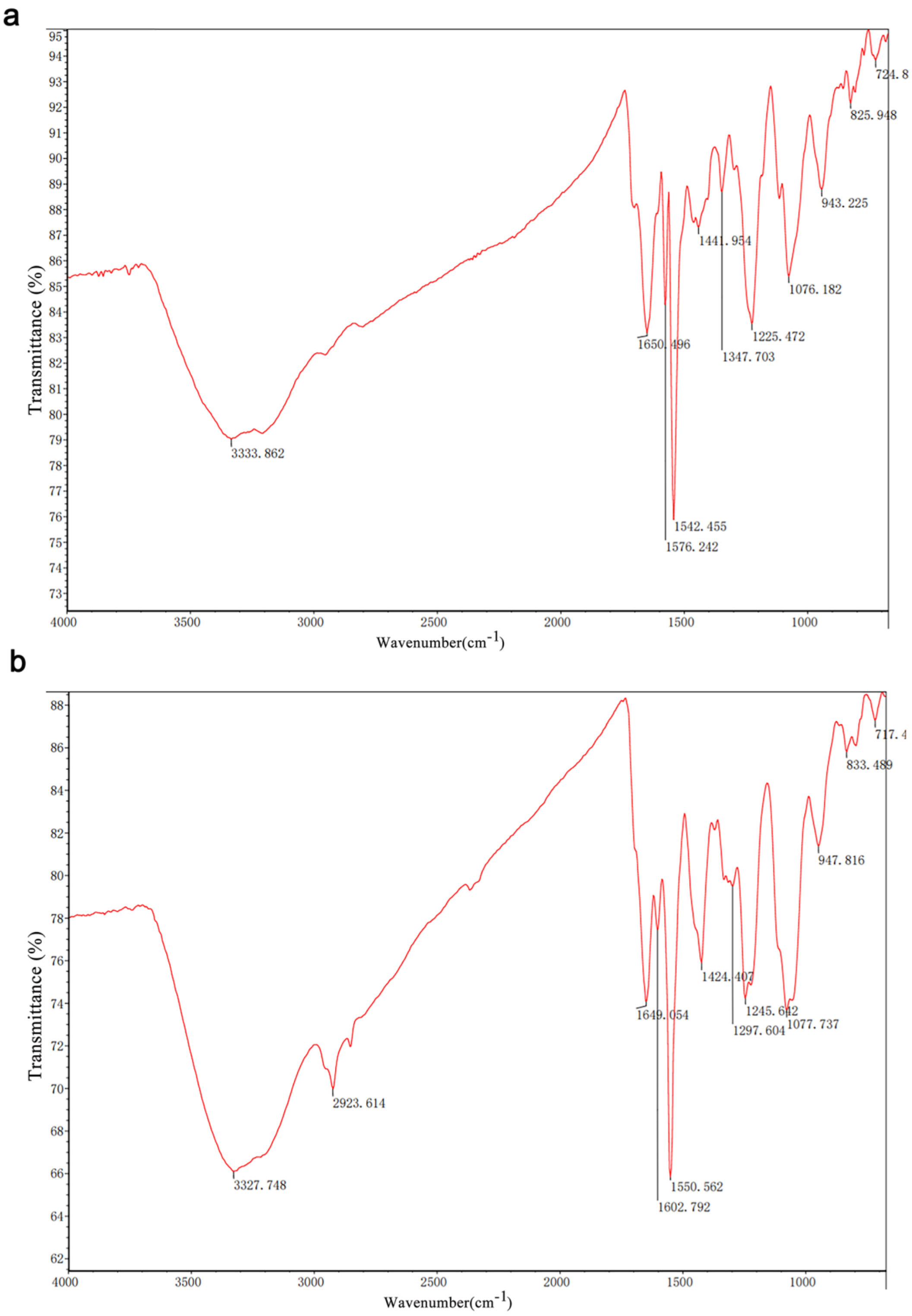

Supplementary Figure 10. IR spectroscopy analyses of the commercial FAD (a) and the purified 6-hydorxy-FAD (b).

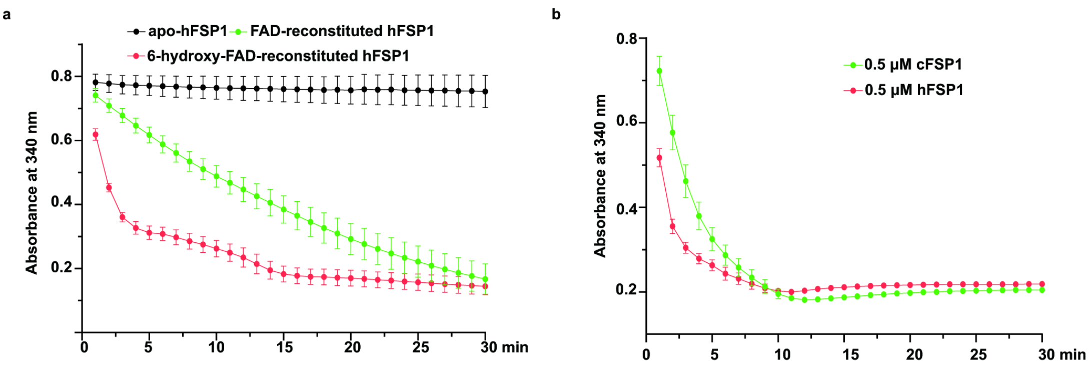

Supplementary Figure 11 NADH oxidation assay (340 nm) of the reconstituted hFSP1 proteins (0.2 uM) as indicated (**a**), and 0.5 uM cFSP1 (green) and hFSP1 (red) (**b**). Apo-hFSP1 lacked cofactors and was prepared by removing all cofactors from the recombinant hFSP1, the FAD- or 6-hydroxy-FAD-reconstituted hFSP1 was prepared by adding the corresponding cofactor into apo-hFSP1. Data in **a** and **b** represent the mean ± s.e.m. of three experiments (*n* = 3). Source data are provided as a Source Data file.

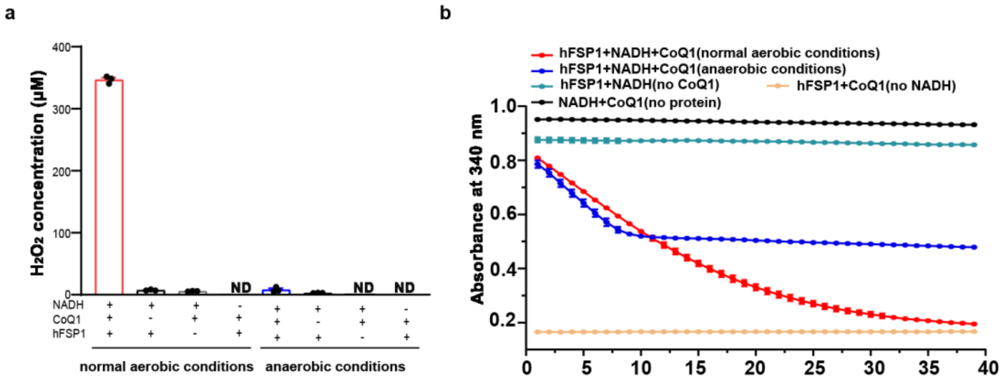

Supplementary Fig 12. Hydrogen peroxide fluorometric assay (**a**) and NADH oxidation assay (**b**) under aerobic or anaerobic conditions as indicated. The reactions contained 50 mM Tris-HCl (pH 8.0)， 250 mM NaCl, hFSP1 (0 or 160 nM), CoQ1 (0 or 200 µM) and NADH (0 or 500 µM). Data in **a** and **b** represent the mean ± s.e.m. of three experiments (*n* = 3). Source data are provided as a Source Data file.

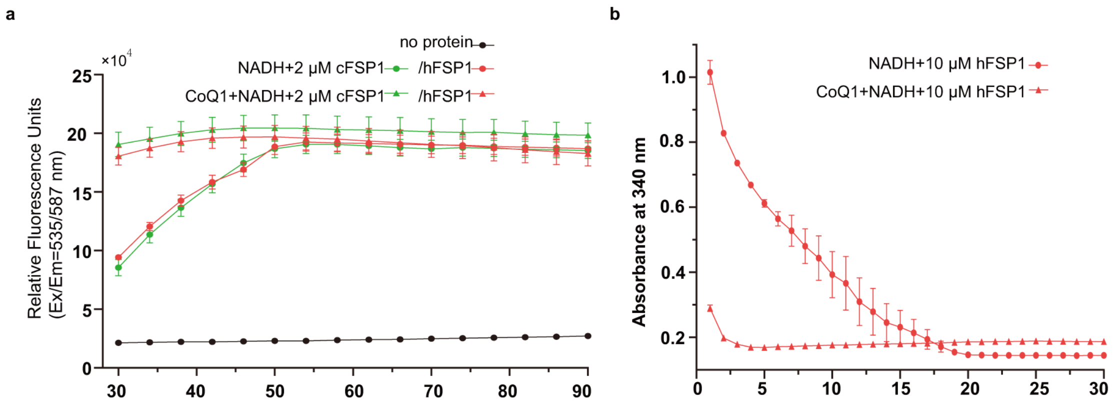

Supplementary Figure 13 Hydrogen peroxide fluorometric assay (**a**) and NADH oxidation assay (**b**) at high enzyme concentration. **a**, Both 2 μM cFSP1 and hFSP1 can produce a large amount of H_2O_2 in the absence of CoQ1 at a lower rate compared to that in the presence of CoQ1. **b**, 10 μM hFSP1 exhibits an obvious NADH oxidation activity in the absence of CoQ, and a more rapid NADH consumption in the presence of CoQ1, which was basically completed within 2 min. Data in **a** and **b** represent the mean ± s.e.m. of three experiments ($n = 3$). Source data are provided as a Source Data file.

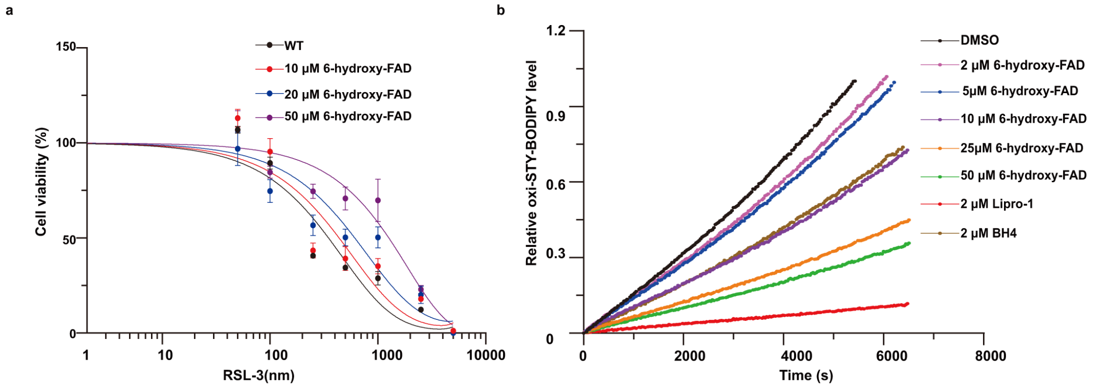

Supplementary Figure 14. Dose response of RSL3-induced cell death of HT1080 wild-type (WT) cells treated with DMSO, different concentrations of 6-hydroxy-FAD (**a**) and Representative autoxidations of STY-BODIPY (1 μM)-embedded liposomes of egg PC lipids (extruded to 100nm, 1mM) initiated by 0.2 mM DTUN and inhibited by DMSO, different concentrations of 6-hydroxy-FAD, 2 μM liproxstatin-1 or BH_4 (**b**). Cell viability in A was assessed using CCK8 assay. Data in **a** represent the mean ± s.e.m. of three experiments ($n = 3$), the experiment in **b** was perfomed only once. Source data are provided as a Source Data file.

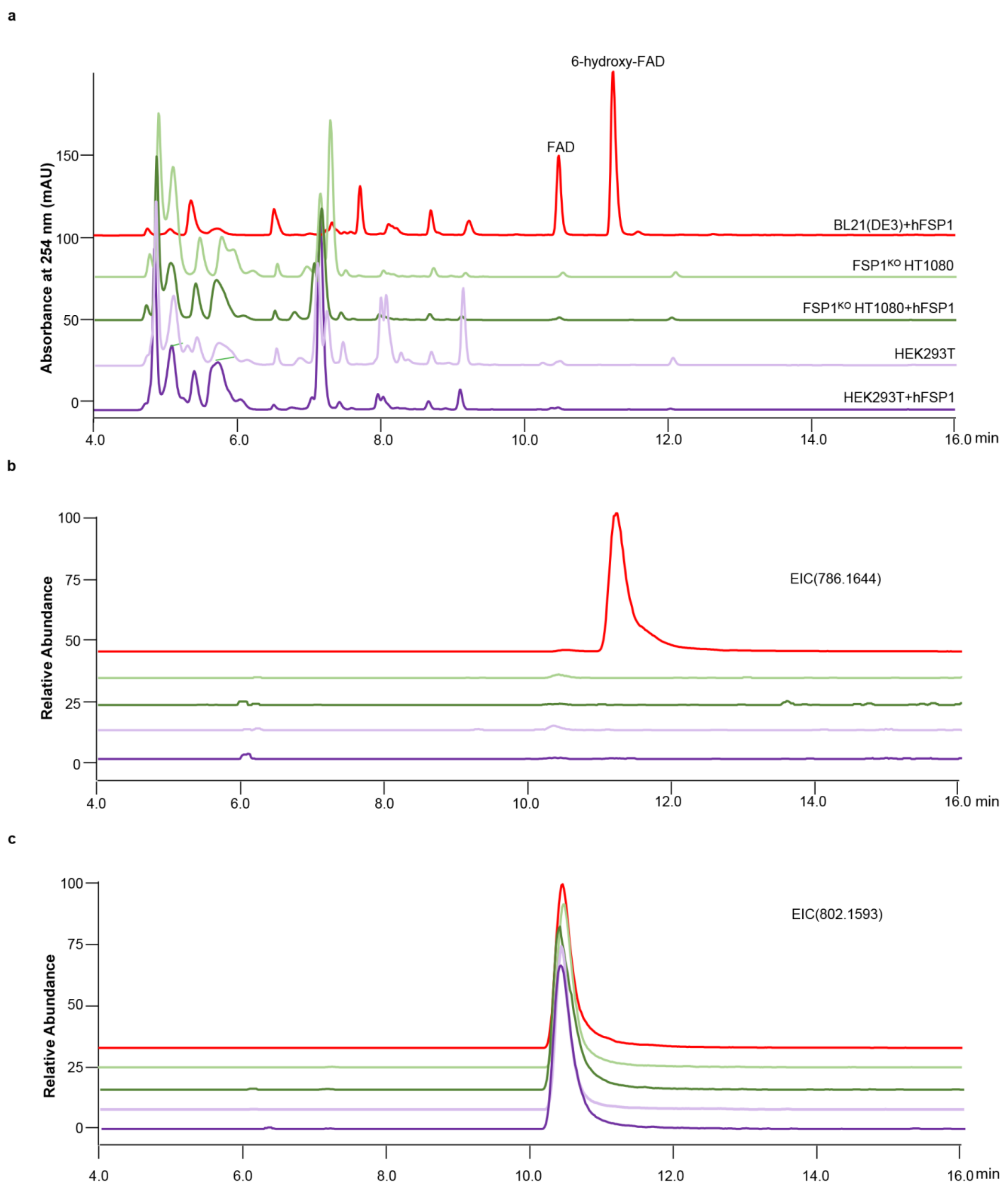

Supplementary Figure 15 HPLC-MS analysis of cell lysates. HPLC chromatograms (**a,** UV 254 nm), MS extracted ion chromatogram (**b,** m/z 786.1644; **c**, m/z 802.1593) of the lysate from BL21(DE3) bacteria overexpressing hFSP1 (red line), FSP1^{KO} HT1080 cells overexpressing empty vector (light green line) or hFSP1 (green line), HEK293T cells overexpressing empty vector (pale purple line) or hFSP1 (purple line). FAD (calculated mass $[M+H]^+$, 786.1644) and 6-hydroxy-FAD (calculalted mass $[M+H]^+$, 802.1593) were eluted at 10.3 min and 11.3 min, respectively. HPLC-MS datasets are available from figshare (https://doi.org/10.6084/m9.figshare.23255453).

Supplementary Table 1. Data Collection and Refinement Statistics

	**cFSP1$^{\Delta N}$** **(PDB 7XPI)**	**cFSP1$^{\Delta N}$** **SeMet**	**cFSP1$^{\Delta N}$-CoQ1** **(PDB 7YTL)**
**Data collection**			
Resolution (Å)	50.00-2.00(2.07-2.00)[a]	50.00-2.37(2.48-2.37)[a]	27.73-2.62 (2.69-2.62)[a]
Wavelength (Å)	0.97911	0.97911	0.97911
Space group	$P\ 6_5\ 2\ 2$	$P\ 6_522$	$P\ 6_522$
Cell dimensions			
a, b, c (Å)	113.376, 113.376, 125.892	113.656, 113.656, 126.087	113.714, 113.714, 125.662
α, β, γ (°)	90, 90, 120	90, 90, 120	90, 90, 120
Total reflections	668985	759636	567977
Unique reflections	32752	20257	14953
Completeness (%)	99.9(100.0)[a]	100.0(100.0)[a]	99.9 (99.7)[a]
Redundancy[a]	20.4 (19.5)[a]	37.5 (36.5)[a]	38.0 (37.4)[a]
$I/\sigma\ (I)$	62.1 (10.4)[a]	43.6 (8.3)[a]	24.2 (2.3)[a]
CC(1/2)(%)	99.3 (98.9)[a]	99.9 (97.9)[a]	100.0 (94.9)[a]
$\boldsymbol{R}_{merge}$[b]	0.067 (0.410)[a]	0.168 (0.724)[a]	0.080 (0.600)[a]
**Refinement**			
Protein copies per ASU	1		1
Resolution（Å）	37.11-2.00		27.73- 2.62
No. reflections	32504		14898
$\boldsymbol{R}_{work}/\boldsymbol{R}_{free}$ (%)[c]	20.00/23.24		20.72/25.82
R.m.s.deviations			
Bond lengths (Å)	0.008		0.009
Bond angles (°)	1.063		1.152
No. atoms			
Protein	2783		2783
Ligand	53		71
Water	264		25
B-factor (Å^2)			
Protein	38.32		62.53
Ligand	26.26		52.83
Water	40.84		51.71
Ramachandran plot[d]			
Favored (%)	97.51		96.40
Allowed (%)	2.49		3.60

Outliers(%)	0.00	0.00

[a] Numbers in parentheses represent the highest resolution shell.

[b] $R_{merge} = \sum_{hkl}\sum_{i}|I_i(hkl) - \langle I(hkl)\rangle| / \sum_{hkl}\sum_{i} I_i(hkl)$, where $\langle I(hkl)\rangle$ is the mean of the observations $I_i(hkl)$ of reflection hkl.

[c] R_{work} and R_{free}: the formula for both is $\sum_{hkl}||F_0| - |F_C|| / \sum_{hkl}|F_0|$, where F_0 and F_C are the observed and calculated structure factors, respectively. R_{work} is calculated for the working set, whereas R_{free} is calculated for the test set (5 % randomly selected data).

[d] As calculated by MolProbity.

Supplementary Table 2. NADH oxidation activity of cFSP1, hFSP1 wild-type and mutants[a]

Function role	cFSP1 mutation site	NADH oxidation (%)[b]
	Wild type	100 ± 2.8
FAD binding	D40A	5.2 ± 0.9
	R42A	28.2 ± 2.3
	V81H	3.4 ± 0.3
	K117A	ND[c]
	D284A	4.2 ± 2
	K292A/M293A/Y295A	ND
	Y343A	53.2 ± 1.5
	Y343F	13.8 ± 1.8
	K354A	40.2 ± 1.4
FAD hydroxylation	R53A	ND
	E155A	36.2 ± 4.5
NADH binding	G150L	15.6 ± 0.7
	H173F	15.5 ± 1.2
ubiquinone binding	Y295A	100 ± 0.4
	L328S	100 ± 3.4
	L329S	40.1 ± 0.7
	L348N	82.5 ± 1.7
	F359A	52.3 ± 2.5
	F359Y	12.6 ± 1.6

C-terminal domain	Δ319-373	ND
	Δ359-373	ND
**Function role**	**hFSP1 mutation site**	**NADH oxidation (%)[d]**
	Wild type	100 ± 4.8
FAD hydroxylation	E156A	ND
CoQ binding	L330S	ND
	F360Y	65.9 ± 0.2
C-terminal domain	Δ317-373	ND
	Δ360-373	ND

[a] In order to simply compare the NADH oxidation activity, we chose to compare the initial activity that was derived from the decrease in the absorbance at 340 nm within five minutes of the assay (reaction rate basically did not change within 5 min). Data represent the mean ± s.e.m. of three experiments ($n = 3$). Source data are provided as a Source Data file.

[b] The corresponding NADH oxidation activity of cFSP1 wild type was taken as 100.0%. The concentration of cFSP1 wild type and mutants in the assay is 0.5 μM.

[c] ND: not detected.

[d] The corresponding NADH oxidation activity of hFSP1 wild type was taken as 100.0%. The concentration of hFSP1 wild type and mutants in the assay is 0.2 μM.

Supplementary Table 3. Kinetic values for FSP1 proteins in aerobic or anaerobic condition

	NADH		NADPH		CoQ[1]		CoQ[2]		CoQ[3]	
	K_m (μM)	k_{cat} (s^{-1})	K_m (μM)	k_{cat} (s^{-1})	K_m (μM)	k_{cat} (s^{-1})	K_m (μM)	k_{cat} (s^{-1})	K_m (μM)	k_{cat} (s^{-1})
**hFSP1**	28.04	2.93	23.01	3.00	16.21	4.41	18.15	4.69	8.99	8.25
**cFSP1**	25.94	1.54	27.18	1.51	17.42	2.54	31.27	3.14		
**6-hydroxy-FAD-reconstitued-hFSP1[4]**	57.36	7.84			24.98	10.58				
**FAD-reconstitued-hFSP1**	19.14	0.98			4.72	1.88				

The reactions contained 50 mM Tris-HCl (pH 8.0), 250 mM NaCl, FSP1 proteins at a constant final concentration (50 nM cFSP1, 25 nM hFSP1, 25 nM 6-hydroxy-FAD-reconstituted hFSP1 or 50 nM FAD-reconstituted hFSP1 in aerobic condition, or 10 nM hFSP1 in anaerobic condition), either with 400 μM CoQ1 and varied NAD(P)H concentration or with 500 μM NAD(P)H and varied CoQ1 concentration. The Kinetic values of NAD(P)H were measured in aerobic condition. Source data are provided as a Source Data file.

1 Measured with 500 μM NADH in aerobic condition.

2 Measured with 500 μM NADPH in aerobic condition.

3 Measured with 500 μM NADH in anaerobic condition.

4 The kinetic values maybe not accurate enough because the initial enzyme velocity may decrease during the detection time.

Supplementary Table 4. Primers used in this paper

Prime name	Sequence
hFSP1 F BamHI	5’-CAGGATCCATGGGTAGCCAGGTTAGCGT-3’
hFSP1 R XhoI	5’-GACTCGAGTCATGGTGGGCTTTGAC-3’
hFSP1 316 R XhoI	5’-GACTCGAGTCAACGCTGTTTAACGCTA-3’
hFSP1 359 R XhoI	5’-GACTCGAGTCACAGGTCACGGCTTTT-3’
hFSP1 (E156A) F	5’-GGTAGCGCAGGTGTGGCGATGGCGGCAGAGATAA-3’
hFSP1 (E156A) R	5’-TTTATCTCTGCCGCCATATTCACACCTGCGCTACCAC-3’
hFSP1 (L330S) F	5’-GCCCTGACCTTCCTGTCGAGCATGGGTCGCAA-3’
hFSP1 (L330S) R	5’-TTGCGACCCATGCTCGACAGGAAGGTCAGGGC-3’
hFSP1 (F360Y) F	5’-CAAAAGCCGTGACCTGTACGTGAGCACTAGC-3’
hFSP1 (F360Y) R	5’-GCTAGTGCTCACGTACAGGTCACGGCTTTTG-3’
cFSP1 F NdeI	5’-CACATATGGGCAGCCGTCTGAGCGT-3’
cFSP1$^{\Delta N}$ F NdeI	5’-CACATATGCGTGTTGTGATTGTTGGCGGT-3’
cFSP1 R XhoI	5’-TACTCGAGGCTTGGCATCGGCTGA-3’
cFSP1 318 R XhoI	5’-GTGGTGGTGGTGGTGCTCGAGGCTTGGCATCGGCTGA-3’
cFSP1 358 R XhoI	5’-GTGGTGGTGGTGGTGCTCGAGCAGATCGCGGCTCTTT-3’
cFSP1 (D40A) F	5’-TCCATTTGTTCTGGTTGCTATGCGCGATGCCTTTC-3’
cFSP1 (D40A) R	5’-GAAAGGCATCGCGCATAGCAACCAGAACAAATGGA-3’
cFSP1 (R42A) F	5’-CATTTGTTCTGGTTGATATGGCCGATGCCTTTCACCATAATG-3’
cFSP1 (R42A) R	5’-CATTATGGTGAAAGGCATCGGCCATATCAACCAGAACAAATG-3’
cFSP1 (R53A) F	5’-TAATGTTGCAGCTCTGGCCGCAAGCGTGGAAAGC-3’
cFSP1 (R53A) R	5’-GCTTTCCACGCTTGCGGCCAGAGCTGCAACATTA-3’
cFSP1 (V81H) F	5’-CGGTGATAGCTTTCGTCAAGGTAAACATGTTGGCATCGATCCG-3’
cFSP1 (V81H) R	5’-CGGATCGATGCCAACATGTTTACCTTGACGAAAGCTATCACCG-3’
cFSP1 (K117A) F	5’-GCGACGGTCCGTTCCCAGGTGCATTCAATAAGGTTATTGATAT-3’
cFSP1 (K117A) R	5’-ATATCAATAACCTTATTGAATGCACCTGGGAACGGACCGTCGC-3’
cFSP1 (E155A) F	5’-GCAGCTGGTGTGGCAATGGCGGCCGAA-3’
cFSP1 (E155A) R	5’-TTCGGCCGCCATTGCCACACCAGCTGC-3’
cFSP1 (G150L) F	5’-CGCATTCTGGTGGTTGGTGGTCTTGCAGCTGGTGT-3’
cFSP1 (G150L) R	5’-ACACCAGCTGCAAGACCACCAACCACCAGAATGCG-3’
cFSP1 (H173F) F	5’-CAAAGAGGTTACACTGATCTTTAGCAAAATCGCGCTGGCT-3’
cFSP1 (H173F) R	5’-AGCCAGCGCGATTTTGCTAAAGATCAGTGTAACCTCTTTG-3’
cFSP1 (D284A) F	5’-TATTTATGCGATCGGTGCCTGCGCGAATCTGAAGG-3’
cFSP1 (D284A) R	5’-CCTTCAGATTCGCGCAGGCACCGATCGCATAAATA-3’
cFSP1 (Y295A) F	5’-AATCTGAAGGAACCAAAAATGGCTGCTCATGCGGAGCTGC-3
cFSP1 (Y295A) R	5’-GCAGCTCCGCATGAGCAGCCATTTTTGGTTCCTTCAGATT-3
cFSP1(K292A/M293A/Y295A) F	5’GGTGACTGCGCGAATCTGAAGGAACCAGCAGCGGCTGCTCATGCGGAGCTGCATGC-3
cFSP1(K292A/M293A/Y295A) R	5’GCATGCAGCTCCGCATGAGCAGCCGCTGCTGGTTCCTTCAGATTCGCGCAGTCACC-3
cFSP1 (L328S) F	5’-CAACCTGGCAGCCTGACCTTTAGTCTGAGCATGGGCAAAAAT-3
cFSP1 (L328S) R	5’-ATTTTTGCCCATGCTCAGACTAAAGGTCAGGCTGCCAGGTTG-3
cFSP1 (L329S) F	5’-CTGGCAGCCTGACCTTTCTGAGTAGCATGGGCAAAAATGATGG-3’

cFSP1 (L329S) R	5’-CCATCATTTTTGCCCATGCTACTCAGAAAGGTCAGGCTGCCAG-3’
cFSP1 (Y343A) F	5’-GTGTTGGCCAAGTTAAAGGTGCCTATGTTGGTCATCTGCTGG-3
cFSP1 (Y343A) R	5’-CCAGCAGATGACCAACATAGGCACCTTTAACTTGGCCAACAC-3
cFSP1 (Y343F) F	5’-GTTGGCCAAGTTAAAGGTTTCTATGTTGGTCATCTGCTG-3’
cFSP1 (Y343F) R	5’-CAGCAGATGACCAACATAGAAACCTTTAACTTGGCCAAC-3’
cFSP1 (L348N) F	5’-CCAAGTTAAAGGTTACTATGTTGGTCATAATCTGGTGACTATT-3
cFSP1 (L348N) R	5’-AATAGTCACCAGATTATGACCAACATAGTAACCTTTAACTTGG-3
cFSP1 (K354A) F	5’-TCTGCTGGTGACTATTGCAGCGAGCCGCGATCTGTTTG-3
cFSP1 (K354A) R	5’-CAAACAGATCGCGGCTCGCTGCAATAGTCACCAGCAGA-3
cFSP1 (F359A) F	5’-TGCAAAGAGCCGCGATCTGGCTGTTAGCAAAAGCTGGAAA-3’
cFSP1 (F359A) R	5’-TTTCCAGCTTTTGCTAACAGCCAGATCGCGGCTCTTTGCA-3’
cFSP1 (F359Y) F	5’-GCAAAGAGCCGCGATCTGTATGTTAGCAAAAGCTG-3
cFSP1 (F359Y) R	5’-CAGCTTTTGCTAACATACAGATCGCGGCTCTTTGC-3

Supplementary References

1 Jumper, J. et al. Highly accurate protein structure prediction with AlphaFold. *Nature* **596**, 583-589 (2021).

2 Lomize, M. A., Pogozheva, I. D., Joo, H., Mosberg, H. I. & Lomize, A. L. OPM database and PPM web server: resources for positioning of proteins in membranes. *Nucleic acids research* **40**, D370-376 (2012).
