# Supplementary material for: Structural insights into FSP1 catalysis and ferroptosis inhibition
Source: Nat Commun. 2023 Sep 22;14:5933. doi: 10.1038/s41467-023-41626-7 (PMC10516921; doi:10.1038/s41467-023-41626-7)
Supplement: Supplementary file 3 — Reporting Summary [file 41467_2023_41626_MOESM3_ESM.pdf]

nature portfolio

Corresponding author(s): Qihai Wang, Huiqing Yuan, Bo Chu, Deyu Zhu

Last updated by author(s): Aug 26, 2023

Reporting Summary

Nature Portfolio wishes to improve the reproducibility of the work that we publish. This form provides structure for consistency and transparency in reporting. For further information on Nature Portfolio policies, see our Editorial Policies and the Editorial Policy Checklist.

Statistics

For all statistical analyses, confirm that the following items are present in the figure legend, table legend, main text, or Methods section.

n/a	Confirmed	
☐	☒	The exact sample size (*n*) for each experimental group/condition, given as a discrete number and unit of measurement
☐	☒	A statement on whether measurements were taken from distinct samples or whether the same sample was measured repeatedly
☐	☒	The statistical test(s) used AND whether they are one- or two-sided<br>*Only common tests should be described solely by name; describe more complex techniques in the Methods section.*
☒	☐	A description of all covariates tested
☒	☐	A description of any assumptions or corrections, such as tests of normality and adjustment for multiple comparisons
☐	☒	A full description of the statistical parameters including central tendency (e.g. means) or other basic estimates (e.g. regression coefficient) AND variation (e.g. standard deviation) or associated estimates of uncertainty (e.g. confidence intervals)
☐	☒	For null hypothesis testing, the test statistic (e.g. *F*, *t*, *r*) with confidence intervals, effect sizes, degrees of freedom and *P* value noted<br>*Give P values as exact values whenever suitable.*
☒	☐	For Bayesian analysis, information on the choice of priors and Markov chain Monte Carlo settings
☒	☐	For hierarchical and complex designs, identification of the appropriate level for tests and full reporting of outcomes
☒	☐	Estimates of effect sizes (e.g. Cohen's *d*, Pearson's *r*), indicating how they were calculated

Our web collection on statistics for biologists contains articles on many of the points above.

Software and code

Policy information about availability of computer code

Data collection	Blu-Ice System for the macromolecular crystallography beamlines of Shanghai Synchrotron Radiation Facility, ZEN 3.6 for the inverted fluorescence microscope (ZEISS Axio Vert. A1), Dynamics 7.0 for Dynamic light scattering (DLS) assay, OTOF Control 3.2 and Hystar3.2 for HPLC-MS data collection.
Data analysis	HKL3000 v720, XDS (Version Mar. 15, 2019), CCP4 v8.0, RESOLVE v2.13, COOT v0.9.8.1, Pymol v2.5.1, Phenix v1.8.4, PHASER v2.8, ChimeraX v1.4, GraphPad Prism v8.0.0 and v9.1.0, Dynamics v7.0, Compass DataAnalysis v4.2, Adobe ILLustrator 2022 v26.4.1

For manuscripts utilizing custom algorithms or software that are central to the research but not yet described in published literature, software must be made available to editors and reviewers. We strongly encourage code deposition in a community repository (e.g. GitHub). See the Nature Portfolio guidelines for submitting code & software for further information.

Data

Policy information about availability of data

All manuscripts must include a data availability statement. This statement should provide the following information, where applicable:

- Accession codes, unique identifiers, or web links for publicly available datasets
- A description of any restrictions on data availability
- For clinical datasets or third party data, please ensure that the statement adheres to our policy

Coordinates and structure factors files have been deposited in the Protein Data Bank with the accession codes 7XPI [https://doi.org/10.2210/pdb7XPI/pdb]

(cFSP1ΔN) and 7YTL [https://doi.org/10.2210/pdb7YTL/pdb] (cFSP1ΔN-CoQ1 complex). All data generated or analyzed during this study are included in this article and its supplementary information file. Source data for the figures, supplementary tables and figures are provided as a Source Data file, except for the HPLC-MS dataset. Source data are provided with this paper. The HPLC-MS dataset is available from Figshare (https://doi.org/10.6084/m9.figshare.23255453). The protein structures used for analysis in the study are available in the Protein Data Bank under accession codes 4NWZ [https://doi.org/10.2210/pdb4NWZ/pdb], 4G6G [https://doi.org/10.2210/pdb4G6G/pdb], 1M6I [https://doi.org/10.2210/pdb1M6I/pdb], 5KMS [https://doi.org/10.2210/pdb5KMS/pdb].

Human research participants

Policy information about studies involving human research participants and Sex and Gender in Research.

Reporting on sex and gender	The study is about structural biology. No reporting on sex and gender.
Population characteristics	This study is not relevant to population characteristics.
Recruitment	No recruitment.
Ethics oversight	No ethics oversight.

Note that full information on the approval of the study protocol must also be provided in the manuscript.

Field-specific reporting

Please select the one below that is the best fit for your research. If you are not sure, read the appropriate sections before making your selection.

☒ Life sciences ☐ Behavioural & social sciences ☐ Ecological, evolutionary & environmental sciences

For a reference copy of the document with all sections, see nature.com/documents/nr-reporting-summary-flat.pdf

Life sciences study design

All studies must disclose on these points even when the disclosure is negative.

Sample size	No sample size calculation was performed. The sample size was determined based on our previous experiences (Adv Sci (Weinh). 2023, 10(6):e2204006; Nat Cell Biol. 2019, 21(5):579-591; FASEB J. 2018,32(4):2036-2045) to ensure reproducibility. For cell experiments, at least three biological replicates were achieved for statistics.
Data exclusions	No data were excluded.
Replication	The co-IP, SDS-PAGE , FENIX, Gel-filtration assays, micro-FTIR, NMR, HPLC and HPLC-MS analysis were performed only once.<br>The western blots, images and DLS are representative of at least two independent experiments with similar results.<br>NADH consumption assay, cell death analysis and hydrogen peroxide concentration detection were repeated independently three times.
Randomization	Randomization was not applicable because our structural analysis and biochemical experiments are not experiments to examine effects on different populations, such as animal studies or clinical trials.
Blinding	Blinding was not applicable because the study is not an animal study or clinical trial, and the data were derived from instrument-based measurement and software-based analysis with minimal risk of bias.

Reporting for specific materials, systems and methods

We require information from authors about some types of materials, experimental systems and methods used in many studies. Here, indicate whether each material, system or method listed is relevant to your study. If you are not sure if a list item applies to your research, read the appropriate section before selecting a response.

Materials & experimental systems

n/a	Involved in the study	
☐	☒	Antibodies
☐	☒	Eukaryotic cell lines
☒	☐	Palaeontology and archaeology
☒	☐	Animals and other organisms
☒	☐	Clinical data
☒	☐	Dual use research of concern

Methods

n/a	Involved in the study	
☒	☐	ChIP-seq
☒	☐	Flow cytometry
☒	☐	MRI-based neuroimaging

Antibodies

Antibodies used	Primary antibody: AIFM2/FSP1 antibody (Proteintech, Cat. #20886-1-AP, 1:1000), HSP90 antibody (ZSGB-BIO, Cat. #TA-12, 1:2000), β-actin antibody (ZSGB-BIO, Cat. #TA-09, 1:2000)?FLAG Tag Antibody (Invitrogen, Cat. #PA1-984B, 1:1000) . Myc-Tag (CST, Cat. #2278, 1:1000).<br>Secondary antibody: Peroxidase AffiniPure Goat Anti-Rabbit IgG (H+L) (Jackson ImmunoResearch Laboratories, Code#111-035-003, 1:3000), Peroxidase AffiniPure Goat Anti-mouse IgG (H+L) (Jackson ImmunoResearch Laboratories, Code#115-035-003,1:3000).
Validation	All antibodies used in the study were commercially bought and validated by the manufacturer as stated on their websites. Manufacturers state that the antibodies have been validated for use.<br>AIFM2/FSP1 Polyclonal antibody (Proteintech, Cat#20886-1-AP) was validated by manufacturer on website (https://www.ptgcn.com/products/AIFM2-Antibody-20886-1-AP.htm) and the previous study (PMID: 31634900) for WB. HSP90 antibody (ZSGB-BIO, Cat#TA-12) was validated by manufacturer on website (http://www.zsbio.com/product/TA-12) and the previous study (PMID: 37086405) for WB. β-actin antibody (ZSGB-BIO, Cat#TA-09) was validated by manufacturer on website (http://www.zsbio.com/product/TA-09) and the previous study (PMID: 31235732) for WB. FLAG Tag Antibody (Invitrogen, Cat#PA1-984B) was validated by manufacturer on website (https://www.thermofisher.cn/cn/zh/antibody/product/DYKDDDDK-Tag-Antibody-Polyclonal/PA1-984B) and the previous study (PMID: 35568705) for IP, the previous study (PMID: 36681781) for WB. Myc Tag Antibody (CST, Cat#2278) was validated by manufacturer on website (https://www.cellsignal.cn/products/primary-antibodies/myc-tag-71d10-rabbit-mab/2278) and the previous study (PMID: 34938412) for IP, the previous study (PMID: 37452028) for WB. Secondary antibody: Peroxidase AffiniPure Goat Anti-Rabbit IgG (H+L) (Jackson ImmunoResearch Laboratories, Code#111-035-003 was validated by manufacturer on website (https://www.jacksonimmuno.com/catalog/products/111-035-003) and the previous study (PMID: 35169117) for WB. Secondary antibody: Peroxidase AffiniPure Goat Anti-mouse IgG (H+L) (Jackson ImmunoResearch Laboratories, Code#115-035-003 was validated by manufacturer on website (https://www.jacksonimmuno.com/catalog/products/115-035-003) and the previous study (PMID: 35650266) for WB.

Eukaryotic cell lines

Policy information about cell lines and Sex and Gender in Research

Cell line source(s)	Human fibrosarcoma (HT1080, Cat. #TCHu170) and human embryonic kidney 293T (HEK293T, Cat. #GNHu17) cells were purchased from the Cell Bank of Shanghai Institute of Biochemistry and Cell Biology (Chinese Academy of Sciences, Shanghai, China) (https://www.cellbank.org.cn), a member of World Federation for Culture Collections (WFCC).
Authentication	None of the cell lines used were authenticated.
Mycoplasma contamination	All cell lines were test negative for mycoplasma contamination by PCR.
Commonly misidentified lines (See ICLAC register)	No commonly misidentified cell line was used .
